# Supplementary material for: Home intravenous diuretic administration for heart failure management: A scoping review
Source: PLoS One. 2025 Jan 17;20(1):e0316851. doi: 10.1371/journal.pone.0316851 (PMC11741602; doi:10.1371/journal.pone.0316851)
Supplement: S2 File — (DOCX) [file pone.0316851.s002.docx]

**Supporting File 2**

**S2 File. Sample search strategy in Ovid MEDLINE.**

Ovid MEDLINE: Epub Ahead of Print, In-Process & Other Non-Indexed Citations, Ovid MEDLINE® Daily and Ovid MEDLINE® <1946-Present>​

 ​

1 exp Heart Failure/ or exp Cardiomyopathies/ 240451 ​

2 ((heart or ventric* or cardiac) adj3 (fail* or decompensat*)).tw,kf. 234698 ​

3 cardiomyopath*.tw,kf. 86406 ​

4 1 or 2 or 3 378883 ​

5 exp Infusions, Intravenous/ or exp Administration, Intravenous/ or exp Injections, Intravenous/ 148445 ​

6 (intravenous* or infus* or inject* or bolus* or parenteral).tw,kf. 1374453 ​

7 5 or 6 1414539 ​

8 exp diuretics/ or exp furosemide/ 83116 ​

9 (diuretic* or furosemide or lasix).tw,kf. 52201 ​

10 8 or 9 109424 ​

11 home care services/ or home care services, hospital-based/ or home health nursing/ or home infusion therapy/ or home nursing/ or home environment/ or outpatients/ or exp ambulatory care/ 120328 ​

12 (home* or domicil* or communit* or ambulatory or outpatient*).tw,kf. 1541957 ​

13 11 or 12 1574785 ​

14 4 and 7 and 10 and 13
