# Supplementary material for: Home intravenous diuretic administration for heart failure management: A scoping review
Source: PLoS One. 2025 Jan 17;20(1):e0316851. doi: 10.1371/journal.pone.0316851 (PMC11741602; doi:10.1371/journal.pone.0316851)
Supplement: Attachment — Submitted filename: Response to Reviewers_27NOV2024.docx [file pone.0316851.s003.docx]

Response to Reviewers re. PLOS ONE: PONE-D-24-43583

Title of Article: Home intravenous diuretic administration for heart failure management: A scoping review.

November 27, 2024

Dear Dr. Lionetti and Reviewers,

Thank you for your review and feedback of our manuscript. We appreciate the opportunity to provide you with a resubmission to address your comments and concerns. Please see below for our comments in reference to the reviewer comments.

| **Reviewer #1** | |
| --- | --- |
| Comments | Responses |
| It would be more useful if there were more information on the results and effectiveness of IV diuretics treatment, if possible. | Thank you for your comment, unfortunately it is difficult to fully understand the outcomes due to variability in study designs, small sample sizes, and diverse patient population. The differences in home care protocols, implementation, and patient monitoring and reporting complicate the standardization of approaches for evaluation or directly compare study results. Furthermore, it was not within the scope of this review to evaluate the outcomes and effectiveness of home IV diuretics as an intervention, but rather understand how the intervention was implemented. The authors have added a comment about this under ‘Limitations’ on page 36:  The dosing approaches observed in these studies included a total daily dose of 40 to 240mg of furosemide, commonly administered via bolus delivery for 1 to 2 weeks (32–34,37,39). Although specific dosing intervals were not always explicitly outlined, standard care practices would typically suggest twice-daily dosing for total daily amounts at or above 80mg, with higher doses up to 120mg twice a day (53). Additionally, the study by Godino et al. reported a mean daily dose of 302.5 ± 176 mg; however, this dosing regimen was delivered through a continuous infusion pump rather than bolus injections, reflecting a variation in administration method that may impact overall efficacy and ability to support this intervention. |
| **Reviewer #2** | |
| Comments | Responses |
| The introduction is deficient in terms of global data, as it presents only the data from Canada as representative | Thank you for your comment. The introduction has been edited to reflect global data of HF burden in the Introduction on page 4:  Heart failure (HF) poses a significant global health burden, with high rates of hospital readmission and mortality affecting patients around the world (1). Despite advances in treatment, many patients experience frequent hospitalizations and declining quality of life, highlighting the urgent need for improved HF management strategies across counties and health systems (1,2,3). The course of chronic HF is troubled by recurrent exacerbation episodes and an overall decline in health status, resulting in frequent hospitalizations and poor quality of life (1,2) Loop diuretics in the form of furosemide are the treatment of choice for symptomatic relief of HF congestion (1,3). However, as the disease progresses, patients become less responsive to oral furosemide and require intravenous (IV) administration to address issues of diuretic resistance and cardio-renal dysfunction (4,5). |
| “The consistency of dosing approaches included a total daily dosing of 40 to 240mg of furosemide by bolus delivery for 1 to 2 weeks, which was the most common (34– 36,39,41).” The administration of 240 mg of furosemide as a bolus is an unlikely course of action. In my view, the discussion lacks a sufficiently analytical summary of the results presented. | Thank you for your comment. Further detail has been added to the discussion about dosing approaches on page 33:  The dosing approaches observed in these studies included a total daily dose of 40 to 240mg of furosemide, commonly administered via bolus delivery for 1 to 2 weeks (32–34,37,39). Although specific dosing intervals were not always explicitly outlined, standard care practices would typically suggest twice-daily dosing for total daily amounts at or above 80mg, with higher doses up to 120mg twice a day (53). Additionally, the study by Godino et al. reported a mean daily dose of 302.5 ± 176 mg; however, this dosing regimen was delivered through a continuous infusion pump rather than bolus injections, reflecting a variation in administration method that may impact overall efficacy and ability to support this intervention. |

Additional edits to the manuscript include the following:

- Change of Headings format to reflect the different levels as outlined in the formatting guideline and sentence casing.
- The Figure title has been reformatted and referenced within the text.
- The Tables have been reformatted and re-ordered within the document to be included directly after the paragraph it is first cited in.
  - Key changes for clarity were made to Table 1, including grouped key characteristics into distinct columns for quick reference, adjusted terminology for uniformity, removed redundant phrases for conciseness while keeping clinical context intact.
- Appendix A, which was the ‘Sample search strategy’ has been removed and resubmitted as a supplemental file, under ‘S1 File’. No alterations have been made to the content.

We thank you for your attention to our submission and look forward to additional feedback.

Kind regards,

Morgan Krauter, MN-NP
